# Supplementary material for: Population genomics of Sitka black-tailed deer supports invasive species management and ecological restoration on islands
Source: Commun Biol. 2022 Mar 10;5:223. doi: 10.1038/s42003-022-03159-5 (PMC8913846; doi:10.1038/s42003-022-03159-5)
Supplement: Supplementary file 2 — Supplementary Information [file 42003_2022_3159_MOESM2_ESM.pdf]

Supplementary Table 1. Mean missing genotypic data percentages and geographic locations of 15 islands (centroids) in Haida Gwaii and their linear distances from Moresby Island.

Island	*Missing data %*	*Latitude*	*Longitude*	*Linear distance from Moresby (km)*
**Bischofs**	5.48	52.575672	-131.561548	3.152
**Burnaby**	10.21	52.381467	-131.327714	0.099
**Faraday**	5.83	52.604820	-131.488162	8.347
**Graham**	3.35	53.527248	-132.422595	0.060
**Hotspring**	1.38	52.576350	-131.440339	8.574
**House**	8.55	52.579844	-131.422818	8.572
**Kunghit**	7.17	52.094273	-131.063761	0.960
**Louise**	14.29	52.948638	-131.778621	0.026
**Lyell**	7.95	52.663058	-131.571116	1.517
**Moresby**	6.93	52.644730	-131.816824	---
**Murchison**	6.27	52.595749	-131.452089	7.044
**Ramsay**	3.78	52.557568	-131.390637	6.118
**Ross**	1.28	52.162139	-131.119845	0.075
**SGang Gwaay**	3.59	52.095328	-131.220495	2.373
**Tanu**	1.03	52.759775	-131.677401	1.585

Supplementary Table 2. Sensitivity analysis performed using STACKS and VCFtools to determine optimal filtering parameters for our neutral SNP dataset; the optimal set of parameters (highlighted in grey), including the minimum percentage of individuals that must contain a locus (*R*) and minimum minor allele frequency (min_maf), was identified to strike a balance between number of individual retained (# Individuals), number of SNPs retained (# SNPs), mean sequence read depth per SNP (Mean Depth per SNP), mean missing data per individuals [Mean Miss per Indv (%)] and genotyping error [Genotyping error % (within/among)].

R	min_maf	# Individuals	# SNPs	Mean Depth per SNP	Mean Miss per Indv (%)	Genotyping error % (within/among)
**0.70**	0.01	178	45595	14.3	14.0	5.67/5.72
	0.02	178	39278	14.8	14.0	
	0.03	181	35604	15.0	14.6	
	0.04	181	32682	15.2	14.6	
	0.05	181	30314	15.5	14.6	
**0.75**	0.01	178	39945	15.1	11.9	
	0.02	181	34226	15.5	12.4	
	0.03	181	30778	15.8	12.4	
	0.04	181	28201	16.0	12.5	
	0.05	181	26136	16.3	12.5	
**0.80**	0.01	181	33377	16.0	10.2	
	0.02	181	28548	16.4	10.3	
	0.03	181	25619	16.8	10.3	
	0.04	181	23442	17.0	10.4	
	0.05	181	21716	17.3	10.4	
**0.85**	0.01	181	25994	17.2	8.1	
	0.02	181	22153	17.7	8.2	
	0.03	181	19884	18.0	8.2	
	0.04	181	18204	18.2	8.3	
	0.05	181	16866	18.5	8.4	
**0.90**	0.01	181	17262	18.8	5.7	
	0.02	181	14541	19.2	5.9	
	0.03	181	12961	19.6	5.9	4.20/3.34
	0.04	181	11787	19.8	6.0	
	0.05	181	10876	20.0	6.1	
**0.95**	0.01	181	7150	20.9	3.2	
	0.02	181	5691	21.3	3.2	
	0.03	181	4942	21.6	3.3	
	0.04	181	4282	21.9	3.4	
	0.05	181	3815	22.1	3.4	3.75/2.12

Supplementary Table 3. Aggregated STRUCTURE results across 10 replicate runs per K value using the entire sample distribution (n=181) implemented using STRUCTURE HARVESTER.

**# *K***	**Reps**	**Mean LnP(*K*)**	**Stdev LnP(*K*)**	**Ln'(*K*)**	**|Ln''(*K*)|**	**Delta *K***
**1**	10	-1738941.0	11.9192	NA	NA	NA
**2**	10	-1642095.2	27.0230	96845.78	82785.91	3063.539510
**3**	10	-1628035.4	45.5943	14059.87	7844.21	172.043474
**4**	10	-1621819.7	364.9444	6215.66	1270.66	3.481791
**5**	10	-1616874.7	708.7565	4945.00	232.42	0.327926
**6**	10	-1611697.3	316.9077	5177.42	37.67	0.118867
**7**	10	-1606557.5	357.6277	5139.75	932.95	2.608719
**8**	10	-1602350.7	862.4163	4206.80	2115.92	2.453479
**9**	10	-1600259.9	5270.4905	2090.88	3261.27	0.618779
**10**	10	-1601430.3	17821.3569	-1170.39	59871.72	3.359549
**11**	10	-1662472.4	206672.5470	-61042.11	131699.33	0.637237
**12**	10	-1591815.1	1408.1555	70657.22	71428.62	50.724953
**13**	10	-1592586.5	5091.2289	-771.40	12600.74	2.474990
**14**	10	-1605958.7	29056.8254	-13372.14	105397.67	3.627295
**15**	10	-1724728.5	134288.694	-118769.81	186425.69	1.388246
**16**	10	-1657072.6	58404.3952	67655.88	75588.67	1.294229
**17**	10	-1665005.4	80734.8847	-7932.79	NA	NA

Supplementary Table 4. Aggregated STRUCTURE results across 10 replicate runs per K value using the entire sample distribution except for SG̲ang Gwaay (n=158) implemented using STRUCTURE HARVESTER.

# *K*	Reps	Mean LnP(*K*)	Stdev LnP(*K*)	Ln'(*K*)	|Ln''(*K*)|	Delta *K*
**1**	10	-1518628.0	5.9093	NA	NA	NA
**2**	10	-1504806.8	26.567	13821.23	7661.97	288.402092
**3**	10	-1498647.5	832.32	6159.27	880.30	1.057646
**4**	10	-1493368.5	271.8259	5278.97	296.70	1.091507
**5**	10	-1488386.3	464.2935	4982.27	890.87	1.918758
**6**	10	-1484294.9	1297.3295	4091.40	1316.80	1.015008
**7**	10	-1478886.7	550.6065	5408.20	1890.20	3.432942
**8**	10	-1475368.7	1748.8975	3518.00	846.97	0.484286
**9**	10	-1472697.6	631.6624	2671.03	4269.17	6.758621
**10**	10	-1474295.8	7155.9077	-1598.13	6295.47	0.879758
**11**	10	-1469598.4	1812.4391	4697.33	4434.77	2.44685
**12**	10	-1469335.9	3353.392	262.57	5974.50	1.781629
**13**	10	-1475047.8	12484.9959	-5711.93	16236.33	1.300468
**14**	10	-1464523.4	558.7136	10524.40	11820.20	21.156098
**15**	10	-1465819.2	1974.3946	-1295.80	2980.47	1.50956
**16**	10	-1464134.5	4192.6449	1684.67	NA	NA

Supplementary Table 5. Contemporary migration rates estimated between islands using BA3-SNPs v.1.1. Source islands are listed across columns at the top. Values in **bold** indicate significant estimates of migration where calculated 95% credible sets (μ ± σ × 1.96) did not include zero. Shaded values along the diagonal indicate the mean proportion of nonmigrants within an island.

To\From	Bischofs	Burnaby	Faraday	Graham	Hotspring	House	Kunghit	Louise	Lyell	Moresby	Murchison	Ramsay	Ross	SGang Gwaay	Tanu
**Bischofs**	0.6979	0.01518	0.0151	0.01508	0.01516	0.01516	0.01514	0.01514	0.0152	**0.08086**	0.01896	0.03552	0.01516	0.01508	0.0153
**Burnaby**	0.01948	0.68626	0.01954	0.01966	0.0196	0.01976	0.01954	0.01958	0.01954	0.05876	0.01946	0.01976	0.0197	0.01968	0.01968
**Faraday**	0.01448	0.01452	0.71006	0.01458	0.01444	0.03808	0.01458	0.01454	0.01448	**0.05792**	0.02902	0.01992	0.01454	0.01446	0.01448
**Graham**	0.01296	0.01286	0.01284	0.7081	0.01284	0.0128	0.01288	0.01274	0.01282	**0.12524**	0.01282	0.0128	0.01288	0.01278	0.0127
**Hotspring**	0.0196	0.0195	0.0196	0.01974	0.68632	0.01958	0.0197	0.01966	0.0196	0.03916	0.01956	0.03932	0.01952	0.01946	0.01968
**House**	0.0153	0.01502	0.01518	0.01526	0.01504	0.75854	0.01508	0.01496	0.01522	0.01514	0.01526	0.0444	0.01528	0.01534	0.01502
**Kunghit**	0.0167	0.01664	0.01676	0.01668	0.0166	0.01664	0.68516	0.01662	0.01666	**0.0984**	0.01664	0.0165	0.0167	0.0167	0.01656
**Louise**	0.01508	0.01506	0.01522	0.01514	0.01506	0.01512	0.01512	0.6971	0.01506	**0.1061**	0.0152	0.01522	0.0151	0.01526	0.01514
**Lyell**	0.01082	0.01072	0.01086	0.01076	0.01076	0.01078	0.0107	0.01084	0.77538	**0.0849**	0.01076	0.01066	0.01074	0.01076	0.01058
**Moresby**	0.0066	0.00638	0.00642	0.00658	0.00634	0.00636	0.00642	0.0063	0.00652	0.91008	0.00646	0.0064	0.00632	0.00648	0.00636
**Murchison**	0.00882	0.00878	0.01768	0.00866	0.00888	0.01756	0.00862	0.00878	0.00878	**0.11904**	0.72736	**0.03068**	0.00882	0.00884	0.0088
**Ramsay**	0.0077	0.00782	0.0079	0.00764	0.00772	0.0156	0.00778	0.00776	0.0075	0.06254	0.01088	0.82616	0.0077	0.00768	0.00766
**Ross**	0.01758	0.01762	0.0175	0.01752	0.01774	0.01756	0.01758	0.01766	0.01748	**0.08716**	0.01754	0.01746	0.68426	0.01756	0.0178
**SGang Gwaay**	0.00878	0.00872	0.00872	0.00876	0.00888	0.00868	0.00878	0.00876	0.009	0.00866	0.00876	0.00894	0.0086	0.8773	0.0086
**Tanu**	0.02078	0.02082	0.0207	0.02074	0.02086	0.02094	0.0208	0.021	0.02078	0.04144	0.0208	0.02102	0.021	0.02084	0.68748
