# Supplementary material for: Population genomics of Sitka black-tailed deer supports invasive species management and ecological restoration on islands
Source: Commun Biol. 2022 Mar 10;5:223. doi: 10.1038/s42003-022-03159-5 (PMC8913846; doi:10.1038/s42003-022-03159-5)
Supplement: Supplementary file 3 — Reporting Summary [file 42003_2022_3159_MOESM3_ESM.pdf]

nature portfolio

Corresponding author(s): Michael Russello

Last updated by author(s): Feb 1, 2022

Reporting Summary

Nature Portfolio wishes to improve the reproducibility of the work that we publish. This form provides structure for consistency and transparency in reporting. For further information on Nature Portfolio policies, see our Editorial Policies and the Editorial Policy Checklist.

Statistics

For all statistical analyses, confirm that the following items are present in the figure legend, table legend, main text, or Methods section.

n/a	Confirmed	
☐	☒	The exact sample size (*n*) for each experimental group/condition, given as a discrete number and unit of measurement
☐	☒	A statement on whether measurements were taken from distinct samples or whether the same sample was measured repeatedly
☒	☐	The statistical test(s) used AND whether they are one- or two-sided<br>*Only common tests should be described solely by name; describe more complex techniques in the Methods section.*
☐	☒	A description of all covariates tested
☐	☒	A description of any assumptions or corrections, such as tests of normality and adjustment for multiple comparisons
☐	☒	A full description of the statistical parameters including central tendency (e.g. means) or other basic estimates (e.g. regression coefficient) AND variation (e.g. standard deviation) or associated estimates of uncertainty (e.g. confidence intervals)
☒	☐	For null hypothesis testing, the test statistic (e.g. *F*, *t*, *r*) with confidence intervals, effect sizes, degrees of freedom and *P* value noted<br>*Give P values as exact values whenever suitable.*
☐	☒	For Bayesian analysis, information on the choice of priors and Markov chain Monte Carlo settings
☒	☐	For hierarchical and complex designs, identification of the appropriate level for tests and full reporting of outcomes
☒	☐	Estimates of effect sizes (e.g. Cohen's *d*, Pearson's *r*), indicating how they were calculated

Our web collection on statistics for biologists contains articles on many of the points above.

Software and code

Policy information about availability of computer code

Data collection	No software used; all DNA sequencing conducted at the McGill University and Génome Québec Innovation Centre.
Data analysis	STACKS v.2.0 bioinformatics workflow (demultiplex and clean DNA sequence reads), bwa mem and ref_map algorithms in BWA (interleave and align data to reference genome and to catalogue and call loci), VCFtools v.0.1.16 (determine optimal filtering parameters via a sensitivity analysis, deviation from Hardy-Weinberg Equilibrium), BayeScan v.2.1 (outlier locus detection), PGDSpider v.2.1.1.5 (input file converter), GenoDive v.3.0 (standard measures of genetic diversity, theta values, analysis of molecular variance, kinship coefficients), SNPRelate R package (principal component analyses), STRUCTURE v.2.3.4 (Bayesian clustering analyses), DISTRUCT v.1.1 (visualize bar plots), BA3-SNPs v.1.1 (posterior estimates of migration rates), 'hierfstat' R package (mean and variance of population assignment indices).

For manuscripts utilizing custom algorithms or software that are central to the research but not yet described in published literature, software must be made available to editors and reviewers. We strongly encourage code deposition in a community repository (e.g. GitHub). See the Nature Portfolio guidelines for submitting code & software for further information.

Data

Policy information about availability of data

All manuscripts must include a data availability statement. This statement should provide the following information, where applicable:

- Accession codes, unique identifiers, or web links for publicly available datasets
- A description of any restrictions on data availability
- For clinical datasets or third party data, please ensure that the statement adheres to our policy

All Illumina raw reads are available from the NCBI sequence read archive (BioProject ID: XXXX); RAD tag sequences and SNP genotypic data are deposited in DRYAD (accession #XXXX)

Field-specific reporting

Please select the one below that is the best fit for your research. If you are not sure, read the appropriate sections before making your selection.

☐ Life sciences ☐ Behavioural & social sciences ☒ Ecological, evolutionary & environmental sciences

For a reference copy of the document with all sections, see nature.com/documents/nr-reporting-summary-flat.pdf

Ecological, evolutionary & environmental sciences study design

All studies must disclose on these points even when the disclosure is negative.

Study description	We used genotyping-by-sequencing (12,947 SNPs) to investigate connectivity and gene flow of invasive Sitka black-tailed deer (n=181) across 15 islands in Haida Gwaii to define eradication units and provide actionable information to guide invasive deer management and ecological restoration within the Gwaii Haanas National Park Reserve, National Marine Conservation Area, and Haida Heritage Site.
Research sample	Sitka black-tailed deer (Odocoileus hemionus sitkensis) ear or muscle tissue samples (n=182 individuals) from 13 islands within Gwaii Haanas, and two islands outside of the national park in greater Haida Gwaii, British Columbia, Canada, representing the diversity of this species within this system.
Sampling strategy	Sitka black-tailed deer ear or muscle tissue samples were collected from 13 islands within Gwaii Haanas, and two islands outside of the national park provided by Parks Canada staff and contractors, as well as from an island-wide hunter sample donation program that was run through the local cutting room.
Data collection	Genomic DNA was extracted from ear tissue using Qiagen® DNeasy® Blood and Tissue Kits following the manufacturer's suggested protocol. Following standardization of DNA quantity (1,000 ng), we constructed four restriction-site associated DNA sequencing (RADseq) libraries using a SbfI RADseq protocol following Baird et al. (2008) as modified in Lemay and Russello (2015). The resulting libraries were sequenced using one full lane each (4 lanes total) of paired-end 125bp sequencing on either an Illumina HiSeq2500 or HiSeq4000 at the McGill University and Génome Québec Innovation Centre.
Timing and spatial scale	Tissue samples were collected between 2017-2019. Genome-wide SNP data collection and analysis were collected in 2020-2021.
Data exclusions	One individual was excluded due to to low sequencing coverage (<5x)
Reproducibility	All raw data are publically available; all packages and parameters for data analysis were described.
Randomization	Individuals were assigned to island of origin for population genetic analyses.
Blinding	n/a

Did the study involve field work? ☒ Yes ☐ No

Field work, collection and transport

Field conditions	Haida Gwaii is an archipelago off the coast of British Columbia, Canada, and is primarily comprised of temperate rainforest.
Location	Haida Gwaii, Britsh Columbia, Canada (52.930995, -132.010060)
Access & import/export	n/a
Disturbance	None; samples were collected as part of on-going invasive species management and sustenance hunting.

Reporting for specific materials, systems and methods

We require information from authors about some types of materials, experimental systems and methods used in many studies. Here, indicate whether each material, system or method listed is relevant to your study. If you are not sure if a list item applies to your research, read the appropriate section before selecting a response.

Materials & experimental systems

n/a	Involved in the study
☒	☐ Antibodies
☒	☐ Eukaryotic cell lines
☒	☐ Palaeontology and archaeology
☐	☒ Animals and other organisms
☒	☐ Human research participants
☒	☐ Clinical data
☒	☐ Dual use research of concern

Methods

n/a	Involved in the study
☒	☐ ChIP-seq
☒	☐ Flow cytometry
☒	☐ MRI-based neuroimaging

Animals and other organisms

Policy information about studies involving animals; ARRIVE guidelines recommended for reporting animal research

Laboratory animals	n/a
Wild animals	Deer were sampled as part of on-going invasive species management and sustenance hunting, which were harvested using a range of approaches including bait station hunting, shoreline hunting, detection dog hunting and aerial hunting
Field-collected samples	Ear or muscle tissue was removed from each individual in the field after death and was either assigned to island of residence or geographic coordinates
Ethics oversight	Parks Canada Agency

Note that full information on the approval of the study protocol must also be provided in the manuscript.
